# Clinical Correlates of Cardiac Conduction in Bipolar Disorder

**DOI:** 10.1192/j.eurpsy.2022.1019

**Published:** 2022-09-01

**Authors:** M. Prieto, A. Carocca, C. Fullerton, A. Hidalgo, J. Diaz, P. San Martin, M. Godoy, M. Nuño, A. De Leon, J. Rodriguez, R. Sanchez, F. Batiz, A. Castillo, A. Cuellar-Barboza, J. Biernacka, M. Frye

**Affiliations:** 1 Universidad de los Andes, Department Of Psychiatry, Santiago, Chile; 2 Clinica Universidad de los Andes, Mental Health Service, Santiago, Chile; 3 Universidad de los Andes, Vicedecanate For Research, Santiago, Chile; 4 Universidad de Chile, Demre, Santiago, Chile; 5 Clinica Universidad de los Andes, Center For Cardiovascular Disease, Santiago, Chile; 6 Private Practice, N/a, Santiago, Chile; 7 Universidad de los Andes, Ciib, Santiago, Chile; 8 Universidad Autonoma de Nuevo Leon, Department Of Psychiatry, Monterrey, Mexico; 9 Mayo Clinic, Psychiatry, Rochester, United States of America

**Keywords:** cardiovascular disease, electrocardiogram, QTc, bipolar disorder

## Abstract

**Introduction:**

Patients with bipolar disorder (BD) have an increased risk for cardiovascular morbimortality. Clinical risk factors, specifically for arrhythmias and sudden cardiac death remain understudied.

**Objectives:**

This study was conducted to assess differences in cardiac conduction among BD patients.

**Methods:**

We included patients with BD in a cross-sectional design, confirmed by structured interview, age 18 through 80. Clinical characteristics were obtained using a structured questionnaire or medical records review. ECG intervals duration and morphology were manually assessed by cardiologists and compared among clinical subgroups using Chi-square, Mann-Whitney, and Kruskall-Wallis tests. Exploratory multivariable linear and logistic regression models were fitted to adjust for potential confounders.

**Results:**

We included 117 patients (60.7% women, 76.9% bipolar I, 50% history of psychosis, 22.6% suicide attempts). We found a significantly longer QTc interval in BD patients with hypertension (difference: 9.5 ms, p=0.006), obesity (difference: 25 ms, p=0.001), and metabolic syndrome (difference: 13 ms, p=0.007). Hypertension remained a significant predictor of longer QTc after adjusting for age, gender, and antipsychotic use (estimate 17.718, p=0.018). We observed a significantly shorter PR interval in women (difference: 6 ms, p=0.029), early age of onset (difference 6 ms, p=0.025), non-users of lithium (difference 4 ms, p=0.002), and early trauma (difference 4 ms, p=0.038). Finally, we identified significant correlations between symptom severity, blood glucose and PR interval (*r*=0.298, p=0.001; *r*=0.278, p=0.003; respectively).

**Conclusions:**

Patients with BD and hypertension may have an increased risk for QTc prolongation. Careful cardiovascular monitoring may be warranted.

**Disclosure:**

No significant relationships.

